# An epigenetic aging analysis of randomized metformin and weight loss interventions in overweight postmenopausal breast cancer survivors

**DOI:** 10.1186/s13148-021-01218-y

**Published:** 2021-12-17

**Authors:** Jamaji C. Nwanaji-Enwerem, Felicia Fei-Lei Chung, Lars Van der Laan, Alexei Novoloaca, Cyrille Cuenin, Harriet Johansson, Bernardo Bonanni, Alan E. Hubbard, Martyn T. Smith, Sheri J. Hartman, Andres Cardenas, Dorothy D. Sears, Zdenko Herceg

**Affiliations:** 1 Department of Emergency Medicine, Gangarosa Department of Environmental Health, Emory University School of Medicine, Emory Rollins School of Public Health, 1518 Clifton Rd. NE, Atlanta, GA 30322 USA; 2 Division of Environmental Health Sciences, School of Public Health and Center for Computational Biology, University of California, Berkeley, Berkeley, CA USA; 3 Epigenomics and Mechanisms Branch, International Agency for Research on Cancer, Lyon, France; 4 Department of Medical Sciences, School of Medical and Life Sciences, Sunway University, Petaling Jaya, Malaysia; 5 Division of Cancer Prevention and Genetics, IEO European Institute of Oncology IRCCS, Via Ripamonti, 435 - 20141 Milan, Italy; 6 College of Health Solutions, Arizona State University, Phoenix, AZ USA; 7 Herbert Wertheim School of Public Health, University of California, San Diego, CA USA; 8 Moores Cancer Center, University of California, San Diego, CA USA; 9 Department Medicine, University of California, San Diego, CA USA

**Keywords:** RCT, DNA methylation age, Biomarkers, GrimAge, PhenoAge

## Abstract

Metformin and weight loss relationships with epigenetic age measures—biological aging biomarkers—remain understudied. We performed a post-hoc analysis of a randomized controlled trial among overweight/obese breast cancer survivors (*N* = 192) assigned to metformin, placebo, weight loss with metformin, or weight loss with placebo interventions for 6 months. Epigenetic age was correlated with chronological age (*r* = 0.20–0.86; *P* < 0.005). However, no significant epigenetic aging associations were observed by intervention arms. Consistent with published reports in non-cancer patients, 6 months of metformin therapy may be inadequate to observe expected epigenetic age deceleration. Longer duration studies are needed to better characterize these relationships.

*Trial Registration*: **Registry Name:** ClincialTrials.Gov.

**Registration Number:** NCT01302379.

**Date of Registration:** February 2011.

**URL:** https://clinicaltrials.gov/ct2/show/NCT01302379

**Supplementary Information:**

The online version contains supplementary material available at 10.1186/s13148-021-01218-y.

## Introduction

Existing evidence suggests that metformin and weight loss interventions may be useful strategies for promoting anti-aging processes including improvements in overall health and lifespan [1]. Still, the relationships of these therapeutic interventions with epigenetic aging (EA) measures—DNA methylation-based biomarkers of biological aging—remain understudied. Along with predicting chronological age, long-term health status, and mortality, EA biomarkers may be helpful in tracking the effectiveness of health interventions such as metformin and weight loss therapy [2, 3]. Nevertheless, randomized trials including EA outcomes in this therapeutic context—and others—remain sparse.

We performed a post-hoc analysis of a randomized controlled trial with a 2 × 2 factorial design (NCT01302379) among overweight/obese postmenopausal breast cancer survivors to examine relationships of metformin and weight loss therapy with nine EA measures [4]. These markers were selected based on their strong associations with health/lifespan and/or their novelty. They also reflect different domains of human biological aging including estimates of mortality, mitosis, and telomere length [5–7]. Hannum, Horvath, and SkinBloodClock epigenetic age are primarily viewed as DNA methylation predictors of chronological age; nonetheless, studies have linked these biomarkers to health status. PhenoAge is a leading biomarker of healthspan, while GrimAge is a biomarker of lifespan. DNAm TL is a DNA methylation-based estimator of telomere length, while mitotic age (MiAge) and epigenetic time to cancer 1/2 (EpiTOC/EpiTOC2) are DNA methylation-based biomarkers of mitotic cell divisions. We hypothesized that metformin and weight loss, in combination or independently, would decelerate epigenetic aging thus reflecting decreased aging-associated disease risk. By performing a comprehensive analysis of EA measures that provide different information on biological aging, we hoped to achieve a more nuanced understanding of metformin and weight loss EA relationships.

## Methods

Study participants (*N* = 333) were randomly assigned to daily metformin, placebo, a weight loss intervention with metformin, or weight loss with placebo for 6 months. Fasting blood samples were collected at baseline and the final 6-month visit. Additional information about the study population, design, and approvals have been previously published [1, 4].

DNA was isolated from buffy coat samples using the Gentra Puregene Blood Kit (Qiagen), and quantified using the Quant-iT™ PicoGreen™ dsDNA Assay Kit (Invitrogen) as per the manufacturers’ protocols. DNA from samples that passed all preliminary quality control steps were processed for bisulfite conversion using 500 ng of each sample with the EZ DNA Methylation Kit (Zymo Research) in accordance with the manufacturer’s instructions.

Given resource constraints, we were only able to perform methylation analyses on 192 of the trial participants. DNA methylation analyses were performed on randomly sampled blood from 192 participants using the Illumina Infinium MethylationEPIC BeadChip. Constrained randomization was conducted to assign one matched pair from each of the four treatment arms to each BeadChip, for a total of 8 samples per chip. The relative positions of the samples were randomized within each BeadChip while keeping matched pairs adjacent to each other. Raw data files were pre-processed, and normalized by functional normalization as implemented in the “minfi” Bioconductor package. Cross-reactive probes [8], probes for which the detection *p*-value exceeded the threshold of 0.01, and probes for which data was missing in > 5% of the samples were excluded, leaving a final data set of 818,493 probes for the samples. Quality control assessments were conducted using the “minfi” Bioconductor package. Raw signal intensities in both green and red channels were consistent across all samples (Additional file 1: Figure S1A and B), and all samples clustered with high signal intensity on both green and red channels (Additional file 1: Figure S1C). Multidimensional scaling (MDS) was applied to evaluate the effect of sample plate, sentrix position, and sentrix ID (Additional file 1: Figure S1D–F) on sample variation, where no clear batch effects were observed. Processed and normalized beta values were then used to calculate measures of EA. EpiTOC/EpiTOC2 and MiAge were calculated using R code from https://doi.org/10.5281/zenodo.2632938 and http://www.columbia.edu/~sw2206/softwares.htm, respectively. The remaining EA measures were calculated using a publicly available calculator (http://dnamage.genetics.ucla.edu).

We first used unadjusted linear mixed effects regression models to examine baseline to end of study differences in each of the nine age-adjusted EA acceleration biomarkers when comparing the intervention arms (weight loss, metformin only, and weight loss plus metformin) to placebo. Models included a random intercept for participants to account for repeated measures. We repeated this analysis using models adjusted for days from randomization to end of study and DNA methylation estimates of leukocyte composition. Finally, we performed unadjusted and adjusted sensitivity analyses comparing high adherence weight loss (≥ 5% weight loss), high adherence metformin (≥ 80% pill adherence), and high adherence weight loss plus high adherence metformin to placebo. All statistical analyses were performed using R Version 3.6.3 (R Core Team, Vienna, Austria).

## Results

**Fig. 1:**
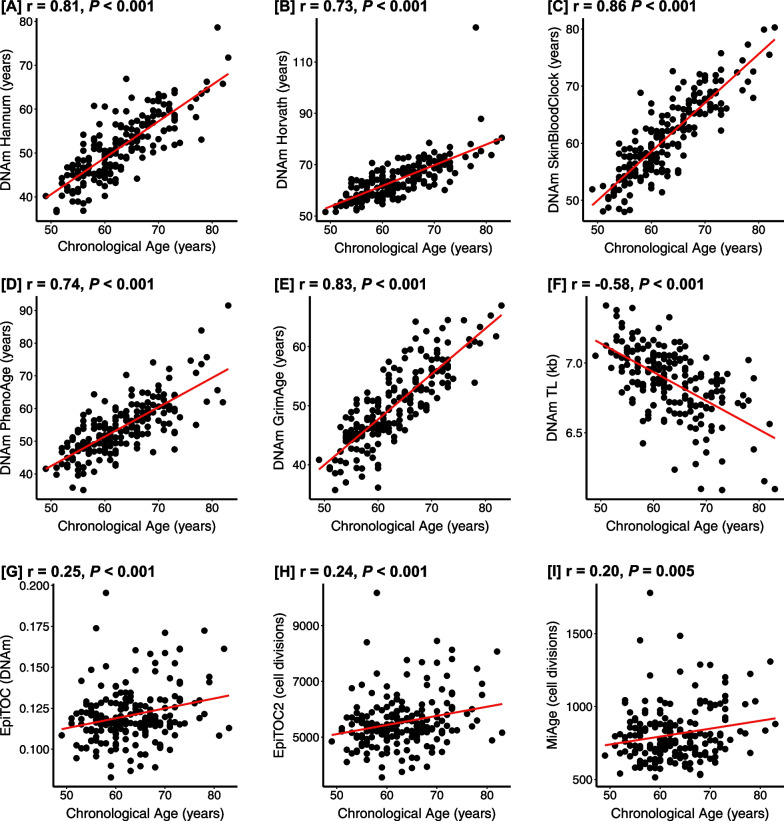
Epigenetic Age and Chronological Age Pearson Correlations. Figure presents the baseline chronological age and epigenetic age correlation coefficients for the study sample (*N* = 192) for DNAmAge Hannum (**A**), DNAmAge Horvath (**B**), DNAmAge SkinBloodClock (**C**), DNAm PhenoAge (**D**), DNAm GrimAge (**E**), DNAm TL (**F**), EpiTOC (**G**), EpiTOC2 (**H**), and MiAge (**I**)

Participant characteristics have been described in previously published work [4]. On average, participants were approximately 63 years of age. In the present analysis, all EA measurements had statistically significant Pearson correlations with chronological age, but the strength of the correlations varied (Fig. 1). The epigenetic mitotic clocks shared the weakest positive correlations with chronological age. Specifically, the MiAge correlation was the weakest (*r* = 0.20, *P* = 0.005). The DNAm SkinBloodClock (*r* = 0.86, *P* < 0.001) followed by DNAm GrimAge (*r* = 0.83, *P* < 0.001) demonstrated the strongest positive chronological age correlations. DNAmTL, as anticipated, was the only measure that was negatively correlated with chronological age (*r* = − 0.58, *P* < 0.001).

In unadjusted intent-to-treat models, when compared to placebo, no treatment arm demonstrated any statistically significant differences or notable trends for any EA marker (Table 1). The results remained null even when intent-to-treat models included adjustments for leukocyte composition and number of days from randomization to the end of the study. Unadjusted and adjusted sensitivity analyses that focused on examining differences between high intervention adherence women and those in the placebo group also did not demonstrate any notable trends for any EA marker. Although weight loss—compared to placebo—was associated with EA in high adherence models, the association was in the opposite direction as expected and would not persist after multiple testing adjustment.

**Table 1:** Estimated difference in epigenetic age biomarkers after 6 months of metformin and weight loss program interventions compared to placebo (*N* = 192)

Aging biomarker models	Weight loss only (*N* = 48)		Metformin only (*N* = 48)		Weight loss plus metformin (*N* = 48)	
	difference in DNA methylation biomarker (95% CI)	*P*	Difference in DNA methylation biomarker (95% CI)	*P*	difference in DNA methylation biomarker (95% CI)	*P*
*EAA Hannum units: years*						
Unadjusted Intent to Treat	1.13 (− 0.46, 2.73)	0.16	0.25 (− 1.35, 1.84)	0.76	1.05 (− 0.55, 2.64)	0.20
Adjusted Intent to Treat	0.98 (− 0.48, 2.44)	0.19	0.47 (− 0.98, 1.91)	0.53	0.45 (− 1.04, 1.95)	0.55
Unadjusted High Adherence*	1.03 (− 0.95, 3.02)	0.30	0.43 (− 1.29, 2.16)	0.62	1.35 (− 0.58, 3.28)	0.17
Adjusted High Adherence*	− 0.04 (− 1.89, 1.81)	0.97	0.60 (− 0.98, 2.19)	0.45	0.61 (− 1.20, 2.43)	0.51
*EAA Horvath units: years*						
Unadjusted Intent to Treat	0.96 (− 0.76, 2.68)	0.27	− 0.20 (− 1.93, 1.52)	0.81	− 0.25 (− 1.97, 1.48)	0.78
Adjusted Intent to Treat	0.74 (− 1.06, 2.53)	0.42	− 0.34 (− 2.11, 1.43)	0.70	− 0.59 (− 2.43, 1.25)	0.53
Unadjusted High Adherence*	− 0.26 (− 2.48, 1.97)	0.82	− 0.21 (− 2.15, 1.72)	0.83	0.38 (− 1.78, 2.55)	0.73
Adjusted High Adherence*	− 0.11 (− 2.49, 2.26)	0.93	− 0.26 (− 2.28, 1.77)	0.80	0.04 (− 2.29, 2.37)	0.97
*EAA SkinBloodClock units: years*						
Unadjusted Intent to Treat	0.81 (− 0.54, 2.15)	0.24	− 0.68 (− 2.03, 0.66)	0.32	0.26 (− 1.08, 1.61)	0.70
Adjusted Intent to Treat	0.86 (− 0.48, 2.19)	0.21	− 0.69 (− 2.01, 0.63)	0.30	0.40 (− 0.96, 1.77)	0.56
Unadjusted High Adherence*	1.01 (− 0.67, 2.68)	0.24	− 0.99 (− 2.45, 0.46)	0.18	0.52 (− 1.10, 2.15)	0.53
Adjusted High Adherence*	0.77 (− 0.90, 2.43)	0.37	− 1.09 (− 2.52, 0.34)	0.13	0.52 (− 1.12, 2.16)	0.53
*Intrinsic EAA (IEAA) units: years*						
Unadjusted Intent to Treat	0.74 (− 0.96, 2.44)	0.39	− 0.22 (− 1.91, 1.48)	0.80	− 0.36 (− 2.06, 1.34)	0.68
Adjusted Intent to Treat	0.74 (− 1.05, 2.53)	0.42	− 0.40 (− 2.17, 1.37)	0.66	− 0.63 (− 2.47, 1.20)	0.50
Unadjusted High Adherence*	− 0.38 (− 2.58, 1.82)	0.73	− 0.16 (− 2.08, 1.75)	0.87	0.12 (− 2.02, 2.26)	0.91
Adjusted High Adherence*	− 0.06 (− 2.45, 2.32)	0.96	− 0.33 (− 2.36, 1.70)	0.75	0.02 (− 2.32, 2.35)	0.99
*Extrinsic EAA (EEAA) units: years*						
Unadjusted Intent to Treat	1.39 (− 0.66, 3.44)	0.18	0.13 (− 1.92, 2.19)	0.90	1.56 (− 0.49, 3.62)	0.13
Adjusted Intent to Treat	1.05 (− 0.55, 2.66)	0.20	0.60 (− 0.99, 2.18)	0.46	0.56 (− 1.08, 2.20)	0.50
Unadjusted High Adherence*	1.57 (− 0.94, 4.08)	0.22	0.42 (− 1.77, 2.60)	0.71	1.79 (− 0.66, 4.23)	0.15
Adjusted High Adherence*	− 0.12 (− 2.15, 1.90)	0.90	0.78 (− 0.95, 2.51)	0.38	0.70 (− 1.29, 2.68)	0.49
*EAA PhenoAge units: years*						
Unadjusted Intent to Treat	1.92 (− 0.30, 4.14)	0.09	0.79 (− 1.43, 3.01)	0.48	0.40 (− 1.82, 2.63)	0.72
Adjusted Intent to Treat	2.02 (0.02, 4.03)	0.05	0.82 (− 1.16, 2.80)	0.41	− 0.12 (− 2.17, 1.93)	0.91
Unadjusted High Adherence*	3.32 (0.56, 6.08)	0.02	1.03 (− 1.37, 3.43)	0.40	0.58 (− 2.11, 3.27)	0.67
Adjusted High Adherence*	2.75 (0.18, 5.32)	0.04	0.98 (− 1.22, 3.17)	0.38	0.48 (− 2.04, 3.00)	0.70
*EAA GrimAge units: years*						
Unadjusted Intent to Treat	0.76 (− 0.61, 2.12)	0.28	− 0.89 (− 2.26, 0.48)	0.20	0.26 (− 1.10, 1.63)	0.70
Adjusted Intent to Treat	0.76 (− 0.58, 2.10)	0.27	− 0.91 (− 2.24, 0.41)	0.18	− 0.39 (− 1.76, 0.98)	0.58
Unadjusted High Adherence*	1.61 (− 0.10, 3.33)	0.07	− 0.65 (− 2.14, 0.85)	0.39	0.45 (− 1.22, 2.12)	0.59
Adjusted High Adherence*	1.38 (− 0.33, 3.10)	0.11	− 0.74 (− 2.21, 0.73)	0.32	− 0.05 (− 1.73, 1.63)	0.95
*DNAm TL Age Adjusted units: kb*						
Unadjusted Intent to Treat	− 0.07 (− 0.14, 0.01)	0.09	− 0.03 (− 0.10, 0.05)	0.52	− 0.02 (− 0.09, 0.06)	0.67
Adjusted Intent to Treat	− 0.04 (− 0.11, 0.02)	0.20	− 0.03 (− 0.10, 0.04)	0.41	0.01 (− 0.06, 0.08)	0.68
Unadjusted High Adherence*	− 0.10 (− 0.20, − 9.5e−4)	0.05	− 2.2e−4 (− 0.09, 0.08)	0.99	− 0.03 (− 0.13, 0.07)	0.53
Adjusted High Adherence*	− 0.06 (− 0.14, 0.03)	0.21	− 0.01 (− 0.08, 0.07)	0.80	0.01 (− 0.08, 0.09)	0.86
*EpiTOC IR units: DNAm*						
Unadjusted Intent to Treat	5.7e−5 (− 5.9e−5, 1.7e−4)	0.34	5.7e−5 (− 5.9e−5, 1.7e−4)	0.34	1.1e−4 (− 5.0e−6, 2.3e−4)	0.06
Adjusted Intent to Treat	1.8e−5 (− 8.1e−5, 1.2e−4)	0.72	4.1e−5 (− 5.6e−5, 1.4e−4)	0.40	5.5e−5 (− 4.5e−5, 1.6e−4)	0.28
Unadjusted High Adherence*	3.0e−5 (− 1.2e−4, 1.8e−4)	0.69	8.9e−5 (− 3.8e−5, 2.2e−4)	0.17	9.0e−5 (− 5.2e−5, 2.3e−4)	0.21
Adjusted High Adherence*	6.6e−6 (− 1.17e−4, 1.3e−4)	0.92	8.5e−5 (− 2.1e−5, 1.9e−4)	0.12	5.1e−7 (− 1.2e−4, 1.2e−4)	0.99
*EpiTOC2 IR units: cell divisions*						
Unadjusted Intent to Treat	3.78 (− 2.29, 9.85)	0.22	2.66 (− 3.41, 8.73)	0.39	5.91 (− 0.16, 11.98)	0.06
Adjusted Intent to Treat	1.43 (− 3.57, 6.44)	0.57	1.85 (− 3.10, 6.81)	0.46	2.73 (− 2.37, 7.84)	0.29
Unadjusted High Adherence*	2.79 (− 4.84, 10.42)	0.47	4.17 (− 2.47, 10.80)	0.22	4.82 (− 2.60, 12.24)	0.20
Adjusted High Adherence*	0.90 (− 5.46, 7.26)	0.78	4.07 (− 1.39, 9.53)	0.14	− 0.18 (− 6.41, 6.05)	0.95
*MiAge IR units: cell divisions*						
Unadjusted Intent to Treat	0.28 (− 0.92, 1.45)	0.66	0.28 (− 0.90, 1.47)	0.64	0.69 (− 0.49, 1.88)	0.25
Adjusted Intent to Treat	− 0.14 (− 1.05, 0.77)	0.77	0.13 (− 0.77, 1.03)	0.78	0.37 (− 0.56, 1.30)	0.44
Unadjusted High Adherence*	0.24 (− 1.22, 1.70)	0.75	0.46 (− 0.81, 1.73)	0.47	0.67 (− 0.75, 2.09)	0.35
Adjusted High Adherence*	− 0.11 (− 1.26, 1.04)	0.85	0.49 (− 0.50, 1.48)	0.33	− 0.11 (− 1.24, 1.02)	0.85

Adjusted models include adjustments for number of days from randomization to end of study and leukocyte abundance/proportions

*Sample sizes for high adherence models are: placebo (*N* = 48), weight loss only (*N* = 23), metformin only (*N* = 36), and weight loss plus metformin (*N* = 25)

EAA = epigenetic age acceleration (residuals of regressing epigenetic age on chronological age)

IR = intrinsic rate of cell divisions (calculated by dividing mitotic clock measurements by chronological age)

## Discussion

Correlations of chronological age with EA demonstrate good to excellent performance of these markers in postmenopausal breast cancer survivors; however, we observe no compelling evidence that leukocyte EA is impacted by 6 months of metformin and/or a weight loss intervention. Still, the reasons for this lack of a statistically significant relationship may be multi-faceted and highly informative for future randomized trials including EA.

It is possible that 6 months is not an adequate amount of time to observe metformin and/or weight loss related leukocyte EA changes. Although the initial trial reported significant changes in blood levels of molecules like insulin and estradiol [4], it is possible that these molecules do not mediate EA changes or that it simply takes longer for these changes to be detectable. This latter assertion is supported by the one existing trial of similarly dosed metformin therapy in combination with growth hormone and dehydroepiandrosterone where significant EA deceleration is only observed after 6 months [9]. Additionally, some EA markers have demonstrated tissue specificity for certain processes. For instance, previous observational studies of obesity were able to identify significant EA acceleration in hepatocytes but not leukocytes from the same subjects [10]. Lastly, the median error of these clocks are larger than one year—a challenge for testing short-term interventions—and our analysis may have been underpowered for this specific EA analysis.

Contrary to our hypothesis, we observed that weight loss—compared to placebo—was associated with accelerated EA in high adherence models. Even if these findings would not persist after multiple testing adjustment, it is worth speculating on the converse nature of this relationship. Although, weight loss is primarily thought to have a beneficial effect on aging and health by improving cardiometabolic and other physiological profiles, there have been reports of the contrary [11, 12]. One important consideration is the coexistence of sarcopenia in older overweight/obese cancer patients [13]. Having diminished lean mass compared to fat mass already places these individuals at risk for frailty/disability [14]. Interventions that focus on weight loss irrespective of the type could potentially lead to further decreases in muscle mass. The exacerbation of this muscle-fat imbalance could be a source of increased morbidity as evidenced by increased biological aging. Still, the phone-based program in this trial had a calorie goal component as well as a moderate-intensity physical activity goal of 300 min/week [1]. Thus, the etiology of this converse relationship in this trial merits further investigation.

In conclusion, randomized trials including EA remain critical for defining the clinical utility of these biomarkers. Intervention duration, characterizing muscle versus fat specific impacts of diet/weight loss interventions, and best matching tissues of EA measurement to biological processes of interest—when possible—remain important considerations in designing future EA randomized trials.

### Supplementary Information

**Additional file 1**.** Supplementary Figure 1**. Quality control and the association between technical parameters and DNA methylation values. Boxplots showing the spread of log2 intensities in (A) green and (B) red channels across the samples analyzed. (C) QC plot of log2 median intensities in the methylated (Meth) and unmethylated (Unmeth) channels. The cut-off for acceptable sample quality is denoted by the dashed diagonal line and demarcates the points where the average of red and green channel log2 median intensities is 10.5. Multidimensional scaling plots of the top 2000 most variable probes in the filtered dataset colored by (D) sample plate, (E) sentrix position and (F) sentrix ID.

## Data Availability

The datasets used and/or analyzed during the current study are not publicly available but inquiries can be made to the senior authors.

## Abbreviations

DNAm TL: DNAm telomere length
EA: Epigenetic aging
EpiTOC: Epigenetic time to cancer
EpiTOC2: Epigenetic time to cancer 2
MiAge: Mitotic age
