# Supplementary material for: An epigenetic aging analysis of randomized metformin and weight loss interventions in overweight postmenopausal breast cancer survivors
Source: Clin Epigenetics. 2021 Dec 17;13:224. doi: 10.1186/s13148-021-01218-y (PMC8684118; doi:10.1186/s13148-021-01218-y)
Supplement: Supplementary file 1 — Additional file 1. Supplementary Figure 1. Quality control and the association between technical parameters and DNA methylation values. Boxplots showing the spread of log2 intensities in (A) green and (B) red channels across the samples analyzed. (C) QC plot of log2 median intensities in the methylated (Meth) and unmethylated (Unmeth) channels. The cut-off for acceptable sample quality is denoted by the dashed diagonal line and demarcates the points where the average of red and green channel log2 median intensities is 10.5. Multidimensional scaling plots of the top 2000 most variable probes in the filtered dataset colored by (D) sample plate, (E) sentrix position and (F) sentrix ID. [file 13148_2021_1218_MOESM1_ESM.pdf]

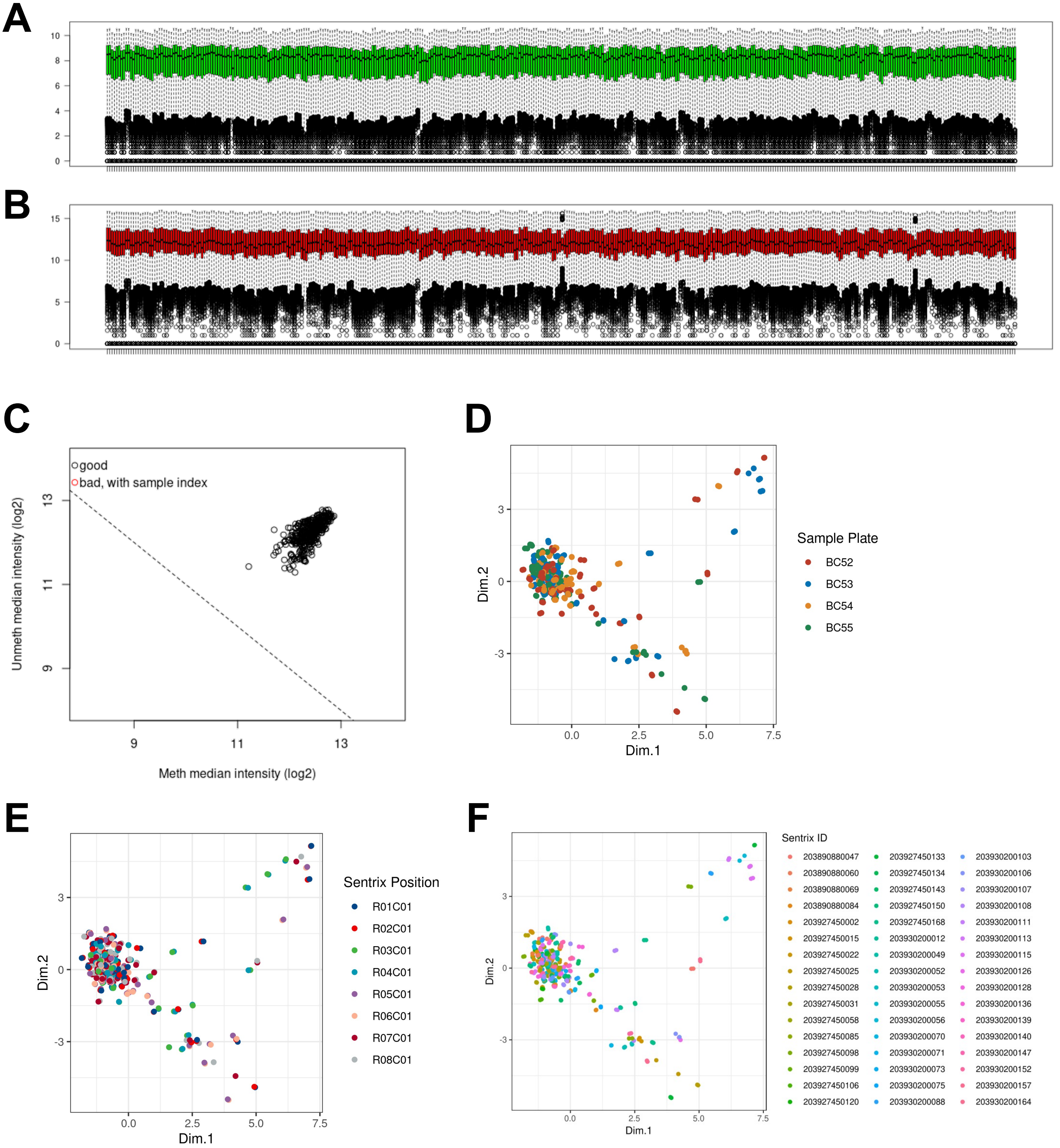

Supplementary Figure 1. Quality control and the association between technical parameters and DNA methylation values. Boxplots showing the spread of $\log_2$ intensities in **(A)** green and **(B)** red channels across the samples analyzed. **(C)** QC plot of $\log_2$ median intensities in the methylated (Meth) and unmethylated (Unmeth) channels. The cut-off for acceptable sample quality is denoted by the dashed diagonal line and demarcates the points where the average of red and green channel $\log_2$ median intensities is 10.5. Multidimensional scaling plots of the top 2000 most variable probes in the filtered dataset colored by **(D)** sample plate, **(E)** sentrix position and **(F)** sentrix ID.
